# A novel real-time PCR assay for quantitative detection of *Campylobacter fetus* based on ribosomal sequences

**DOI:** 10.1186/s12917-016-0913-3

**Published:** 2016-12-15

**Authors:** Gregorio Iraola, Ruben Pérez, Laura Betancor, Ana Marandino, Claudia Morsella, Alejandra Méndez, Fernando Paolicchi, Alessandra Piccirillo, Gonzalo Tomás, Alejandra Velilla, Lucía Calleros

**Affiliations:** 1 Sección Genética Evolutiva, Facultad de Ciencias, Iguá 4225, Montevideo, 11400 Uruguay; 2 Unidad de Bioinformática, Institut Pasteur Montevideo, Montevideo, Uruguay; 3 Laboratorio de Bacteriología, Unidad Integrada INTA-Universidad Nacional de Mar del Plata, Balcarce, Argentina; 4 Departamento de Bacteriología y Virología, Instituto de Higiene, Facultad de Medicina, Universidad de la República, Montevideo, Uruguay; 5 Dipartimento di Biomedicina Comparata e Alimentazione, Università degli Studi di Padova, Padova, Italy

**Keywords:** *Campylobacter fetus*, Molecular detection, Real-time PCR

## Abstract

**Background:**

*Campylobacter fetus* is a pathogen of major concern for animal and human health. The species shows a great intraspecific variation, with three subspecies: *C. fetus* subsp. *fetus*, *C. fetus* subsp. *venerealis*, and *C. fetus* subsp. *testudinum*. *Campylobacter fetus fetus* affects a broad range of hosts and induces abortion in sheep and cows. *Campylobacter fetus venerealis* is restricted to cattle and causes the endemic disease bovine genital campylobacteriosis, which triggers reproductive problems and is responsible for major economic losses. *Campylobacter fetus testudinum* has been proposed recently based on genetically divergent strains isolated from reptiles and humans. Both *C. fetus fetus* and *C. fetus testudinum* are opportunistic pathogens for immune-compromised humans. Biochemical tests remain as the gold standard for identifying *C. fetus* but the fastidious growing requirements and the lack of reliability and reproducibility of some biochemical tests motivated the development of molecular diagnostic tools. These methods have been successfully tested on bovine isolates but fail to detect some genetically divergent strains isolated from other hosts. The aim of the present study was to develop a highly specific molecular assay to identify and quantify *C. fetus* strains.

**Results:**

We developed a highly sensitive real-time PCR assay that targets a unique region of the *16S* rRNA gene. This assay successfully detected all *C. fetus* strains, including those that were negative for the *cstA* gene-based assay used as a standard for molecular *C. fetus* identification. The assay showed high specificity and absence of cross-reactivity with other bacterial species. The analytical testing of the assay was determined using a standard curve. The assay demonstrated a wide dynamic range between 10^2^ and 107 genome copies per reaction, and a good reproducibility with small intra- and inter-assay variability.

**Conclusions:**

The possibility to characterize samples in a rapid, sensitive and reproducible way makes this assay a good option to establish a new standard in molecular identification and quantification of *C. fetus* species.

**Electronic supplementary material:**

The online version of this article (doi:10.1186/s12917-016-0913-3) contains supplementary material, which is available to authorized users.

## Background

Members of the genus *Campylobacter* are gram-negative epsilon-proteobacteria highly adapted to vertebrate hosts. Most species are pathogens of a wide range of livestock species and have extensive reservoirs in wildlife [1–3].

The species *Campylobacter fetus* shows a remarkable level of intraspecific variation, with three subspecies: *C. fetus* subsp. *fetus*, *C. fetus* subsp. *venerealis*, and *C. fetus* subsp. *testudinum. Campylobacter fetus fetus* and *C. fetus venerealis* are classified on the basis of their mechanisms of transmission, clinical presentations and two key biochemical tests (tolerance to glycine and H_2_S production) [4, 5]. *Campylobacter fetus fetus* infects the intestinal tract of several mammalian species and induces abortion in cattle and sheep [2, 5, 6]. In humans, it is an opportunistic pathogen that mainly infects immune-compromised patients [7, 8]. *Campylobacter fetus venerealis* is a cattle-restricted pathogen with tropism for genital tissues and is the etiological agent of bovine genital campylobacteriosis (BGC), a serious reproductive disease that causes infertility and abortion [9]. *C. fetus venerealis* includes a variant, namely *C. fetus venerealis* biovar intermedius that reacts differently to the H_2_S test and also causes BGC [5]. *Campylobacter fetus testudinum* has been proposed recently to cluster some reptilian and human strains of putative reptilian origin on the basis of notorious genetic divergence from *C. fetus fetus* and *C. fetus venerealis* [10].

Biochemical tests remain as the gold standard for identifying *C. fetus* and differentiating between *C. fetus fetus* and *C. fetus venerealis*, but the fastidious growth requirements and the lack of reliability and reproducibility of some assays [11], due in part to the genetic heterogeneity of some strains, motivated the development of alternative diagnostic methods.

Several studies have endeavored in determining the suitability of different genetic methods for identifying the species *C. fetus* using end-point PCRs. In particular, the multiplex-PCR assay designed by Hum et al. [12] has been vastly used for species identification. Detection of *C. fetus* in this assay is achieved using PCR primers that target signature regions of the *cstA* gene, and *C. fetus venerealis* identification is based on the *parA* gene. However, genetic divergence in the *cstA* gene could prevent their detection by this assay, as occur in reptilian strains, and thus fails as a general diagnostic tool to identify the species [10].

Other assays for *C. fetus* identification were later designed to target additional genes, like *cpn60*, which encodes the universal 60-kDa chaperonin, and *nahE*, which encodes a sodium/hydrogen exchanger protein [13, 14]. The *cpn60* and *nahE* gene-based methods have been updated to real-time PCR assays using different technologies [14–17]. Both real-time assays have been designed to detect *C. fetus* on bovine isolates, and successfully tested on this kind of samples, but may fail to detect some genetically divergent strains, particularly of reptilian origin, which have distinctive nucleotide variants in many genes. Therefore, detection of *C. fetus* can be improved by developing new real-time PCR assays able to detect strains from all subspecies and hosts. These assays should be designed to target highly stable genomic regions that are characteristic for the species. Ribosomal genes are one of the most common DNA regions used to design PCR assays for the identification and detection of microorganisms. The *16S* rRNA gene-targeted molecular tools are widely used as its variability has been thoroughly described in all *Campylobacter* species [18–23]. The sequence of the *16S* rRNA gene is species-specific within the genus and *C. fetus* has several unique nucleotide markers [24, 25]. Moreover, ribosomal genes are homogeneous for *C. fetus* subspecies and have three identical copies per genome allowing a better detection. Despite the obvious advantages of these genes, so far, there is not a real-time PCR assay targeting ribosomal sequences for the specific detection of *C. fetus*.

The aim of the present study was to develop a highly sensitive real-time PCR assay, to detect and quantify *C. fetus* strains.

## Results

Strains were assigned to *C. fetus* and its subspecies using standard bacteriological methods (Table 1). Additionally, we performed the molecular characterization in the same collection of strains (Table 1). The results of bacteriological and molecular classification do not always match, particularly at the subspecies level One bovine (INTA 89/222) and the reptilian isolate (RA8/Italy/2011) were phenotypically identified as *C. fetus* but were negative for the *cstA* gene amplicon that is currently used as a marker for *C. fetus*. The bovine isolate was positive for the subspecies (*C. fetus venerealis*) markers of both tests and the reptilian isolate was negative. The assignment of these isolates to the species *C. fetus* was confirmed by sequencing a fragment of the *16S* rRNA gene, which unequivocally discriminates between *Campylobacter* species and from other bacterial species [21, 24].

**Table 1:** Isolates analyzed, discriminated by host, source, country and year of isolation

Isolate	Host	Source	Country	Year	Phenotypic typing^a^	Multiplex PCR A^b^	Multiplex PCR B^c^	Real-time PCR
A28	Bovine	U	Australia	1978	Cff	Cff	Cff	+
063	Bovine	Prepuce	Uruguay	1980	Cff	Cff	Cff	+
0835	Bovine	U	Uruguay	U	Cff	Cfv	Cff	+
F106	Bovine	U	Uruguay	U	Cff	Cff	Cff	+
71098	Bovine	Fetal abomasal content	Uruguay	1998	Cff	Cff	Cff	+
INTA 97/C1N3^d^	Bovine	Vaginal mucus	Argentina	1997	Cff	Cff	Cff	+
INTA 04/554	Bovine	Fetal abomasal content	Argentina	2004	Cff	Cff	Cff	+
INTA 90/189	Bovine	Fetal lung	Argentina	1990	Cff	Cfv	Cfv	+
INTA 89/222	Bovine	Prepuce	Argentina	1989	Cff	No Cf/Cfv	No Cf/Cfv	+
INTA 01/165	Bovine	Vaginal mucus	Argentina	2001	Cff	Cff	Cff	+
INTA 12/218	Bovine	Fetal abomasal content	Argentina	2012	Cff	Cfv	Cfv	+
INTA 99/801	Bovine	Prepuce	Argentina	1999	Cff	Cff	Cff	+
INTA 01/064	Bovine	Vaginal mucus	Argentina	2001	Cff	Cff	Cff	+
INTA 04/875	Bovine	Vaginal mucus	Argentina	2004	Cff	Cff	Cff	+
INTA 08/328	Bovine	Fetal lung	Argentina	2008	Cff	Cff	Cff	+
INTA 05/622	Bovine	Fetal abomasal content	Argentina	2005	Cff	Cff	Cfv	+
INTA 11/262	Bovine	Fetal abomasal content	Argentina	2011	Cff	Cfv	Cfv	+
INTA 11/295	Bovine	Fetal abomasal content	Argentina	2011	Cff	Cfv	Cfv	+
INTA 11/685A	Bovine	Vaginal mucus	Argentina	2011	Cff	Cfv	Cff	+
INTA 11/685B	Bovine	Fetal abomasal content	Argentina	2011	Cff	Cfv	Cff	+
INTA 11/677	Bovine	Fetal abomasal content	Argentina	2011	Cff	Cff	Cff	+
INTA 11/501	Bovine	Vaginal mucus	Argentina	2011	Cff	Cff	Cff	+
INTA 11/408	Bovine	Fetal abomasal content	Argentina	2011	Cff	Cff	Cff	+
INTA 11/356	Bovine	Fetal abomasal content	Argentina	2011	Cff	Cff	Cfv	+
INTA 11/360	Bovine	Fetal lung	Argentina	2011	Cff	Cfv	Cfv	+
NCTC10354^T^	Bovine	U	England	1962	Cfv	Cff	Cfv	+
D78	Bovine	U	Australia	1978	Cfv	Cfv	Cfv	+
660	Bovine	Fetal abomasal content	Uruguay	2010	Cfv	Cfv	Cfv	+
3726	Bovine	Fetal abomasal content	Uruguay	2010	Cfv	Cfv	Cfv	+
2733	Bovine	Fetal abomasal content	Uruguay	2006	Cfv	Cfv	Cfv	+
2740	Bovine	Fetal abomasal content	Uruguay	2006	Cfv	Cfv	Cfv	+
MCR03	Bovine	Prepuce	Uruguay	2009	Cfv	Cfv	Cfv	+
3837	Bovine	Fetal abomasal content	Uruguay	2010	Cfv	Cfv	Cfv	+
1198	Bovine	U	Uruguay	U	Cfv	Cff	Cfv	+
3598	Bovine	U	Uruguay	U	Cfv	Cff	Cfv	+
2432	Bovine	U	Uruguay	2010	Cfv	Cfv	Cfv	+
2370P	Bovine	Fetal abomasal content	Uruguay	2011	Cfv	Cfv	Cfv	+
2374C	Bovine	Fetal abomasal content	Uruguay	2011	Cfv	Cfv	Cfv	+
27460P	Bovine	Fetal abomasal content	Uruguay	2011	Cfv	Cfv	Cfv	+
INTA 97/608^d^	Bovine	Placenta	Argentina	1997	Cfv	Cfv	Cfv	+
INTA 83/371	Bovine	Vaginal mucus	Argentina	1983	Cfv	Cfv	Cfv	+
INTA 90/264	Bovine	Fetal abomasal content	Argentina	1990	Cfv	Cff	Cfv	+
INTA 05/355	Bovine	Fetal abomasal content	Argentina	2005	Cfv	Cfv	Cfv	+
INTA 95/258	Bovine	Vaginal mucus	Argentina	1995	Cfv	Cff	Cfv	+
INTA 08/382	Bovine	Fetal abomasal content	Argentina	2008	Cfv	Cff	Cfv	+
021	Bovine	U	Australia	1978	Cfvi	Cfv	Cfv	+
INTA 98/BL472	Bovine	Fetal abomasal content	Argentina	1998	Cfvi	Cfv	Cfv	+
INTA 99/541	Bovine	Prepuce	Argentina	1999	Cfvi	Cff	Cfv	+
INTA 97/384	Bovine	Fetal abomasal content	Argentina	1997	Cfvi	Cff	Cfv	+
INTA 98/472	Bovine	Fetal abomasal content	Argentina	1998	Cfvi	Cfv	Cfv	+
INTA 00/305	Bovine	Fetal abomasal content	Argentina	2000	Cfvi	Cff	Cfv	+
INTA 02/146	Bovine	Vaginal mucus	Argentina	2002	Cfvi	Cfv	Cfv	+
INTA 03/596	Bovine	Fetal abomasal content	Argentina	2003	Cfvi	Cff	Cff	+
INTA 07/379	Bovine	Fetal abomasal content	Argentina	2007	Cfvi	Cff	Cfv	+
INTA 06/341	Bovine	Fetal lung	Argentina	2006	Cfvi	Cfv	Cfv	+
H1-UY	Human	Blood	Uruguay	2013	Cf	Cff	Cff	+
HC	Human	Blood	Uruguay	2014	Cf	Cff	Cff	+
70 L	Human	Cerebrospinal fluid	Uruguay	2014	Cf	Cff	Cff	+
70H	Human	Blood	Uruguay	2014	Cf	Cff	Cff	+
RA8/Italy/2011	Turtle	Cloaca	Italy	2011	Cft	No Cf	No Cf	+
RC7	Turtle	Cloaca	Italy	2011	*C. geochelonis*	No Cf	No Cf	-
RC11	Turtle	Cloaca	Italy	2011	*C. geochelonis*	No Cf	No Cf	-
RC20	Turtle	Cloaca	Italy	2011	*C. geochelonis*	No Cf	No Cf	-
INTA 08/209	Bovine	Prepuce	Argentina	2008	*C. sputorum*	No Cf	No Cf	-
CcHB41	Human	Feces	Uruguay	2010	*C. coli*	No Cf	No Cf	*-*
CjHB32	Human	Feces	Uruguay	2010	*C. jejuni*	No Cf	No Cf	*-*
CjCP3	Chicken	Cecal content	Uruguay	2010	*C. jejuni*	No Cf	No Cf	*-*
CcCP60	Chicken	Cecal content	Uruguay	2009	*C. coli*	No Cf	No Cf	*-*
INTA 99/243	U	Vaginal mucus	Argentina	1999	*C. hyointestinalis*	No Cf	No Cf	*-*
NCTC 11562	Pork	U	England	1983	*C. hyointestinalis*	No Cf	No Cf	*-*

*Cft Campylobacter fetus* subsp. *testudinum*, *Cff Campylobacter fetus* subsp. *fetus*, *Cfv Campylobacter fetus* subsp. *venerealis*, *Cfvi Campylobacter fetus* subsp. *venerealis* biovar intermedius, *Cf Campylobacter fetus*, *U* unknown, *ND* not determined

^a^in *C. fetus*, glycine tolerance and H2S production, see text for details

^b^As described in Hum et al. [12]

^c^As described in Iraola et al. [41]

^d^These strains were assayed both starting from a resuspended culture and directly from bovine samples of placenta or vaginal mucus, without a previous isolation step

**Fig. 1:**
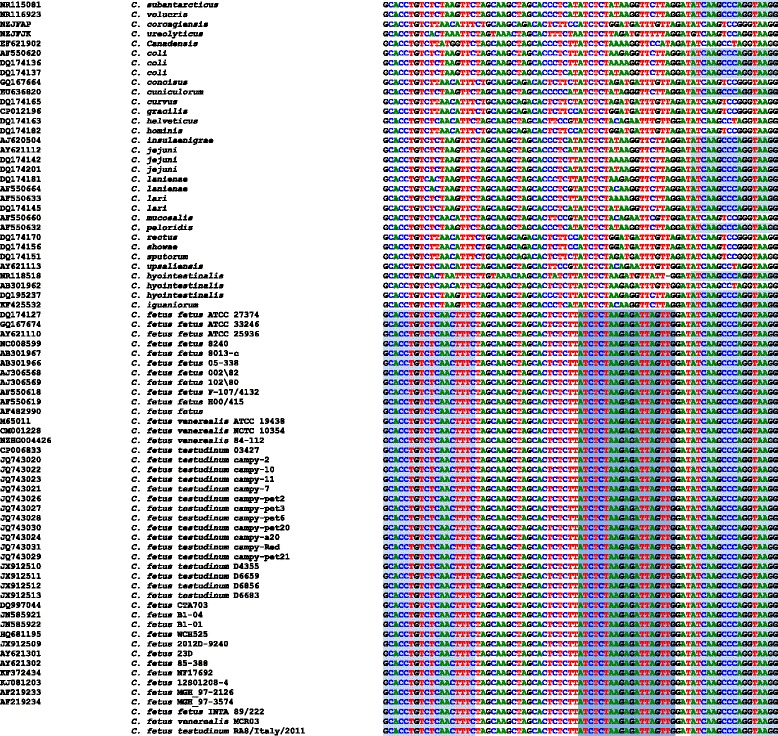
Multiple alignment of partial sequences of *16S* gene obtained from databases. Sequences of all species of the genus from which information is available are shown. The sequences of the primers and probe are shaded

**Fig. 2:**
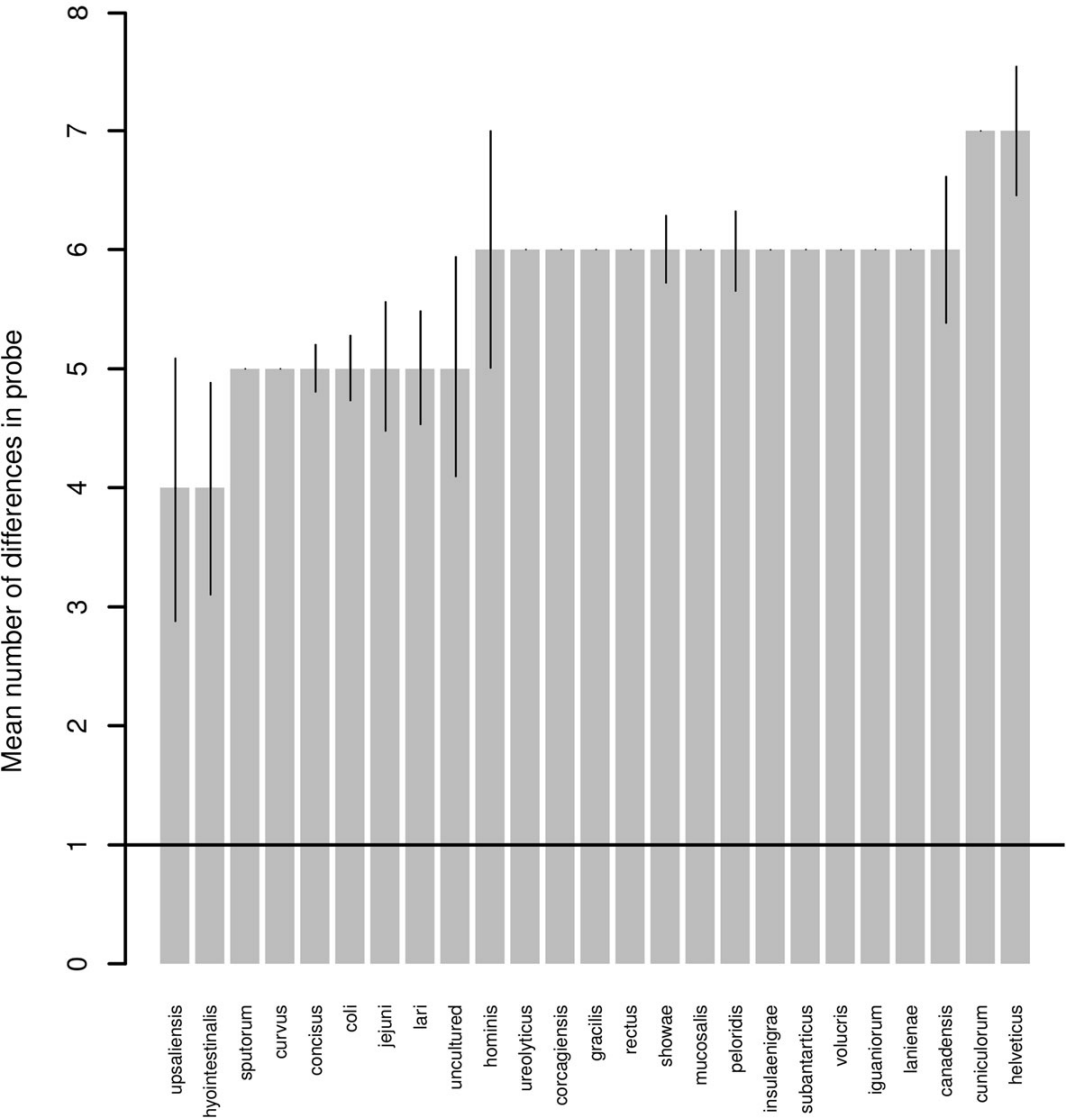
Mean number of differences in probe sequence of non-*C. fetus* species *16S* gene

The 16SPb probe is species specific and has a minimum of one mismatch with a single sequence from *C. hyointestinalis*, and a maximum of nine differences with other *Campylobacter* species (e.g. *C. rectus* and *C. showae*). The forward primer’s sequence is species specific and has a minimum of one and a maximum of four mismatches with other *Campylobacter* species (Figs. 1 and 2, Additional file 1). The reverse primer’s sequence is identical in some *Campylobacter* species but has one or two differences with others. The combination of primers and probe only matches perfectly with the *16S* rRNA gene of *C. fetus.*

All PCR reactions using template DNA from *C. fetus fetus, C. fetus venerealis, C. fetus venerealis* bv. intermedius*,* and *C. fetus testudinum* yielded a VIC signal corresponding to the *C. fetus*-specific probe. This result indicates a 100% clinical sensitivity and 95% confidence interval of 94–100% (Clopper-Pearson interval).

**Fig. 3:**
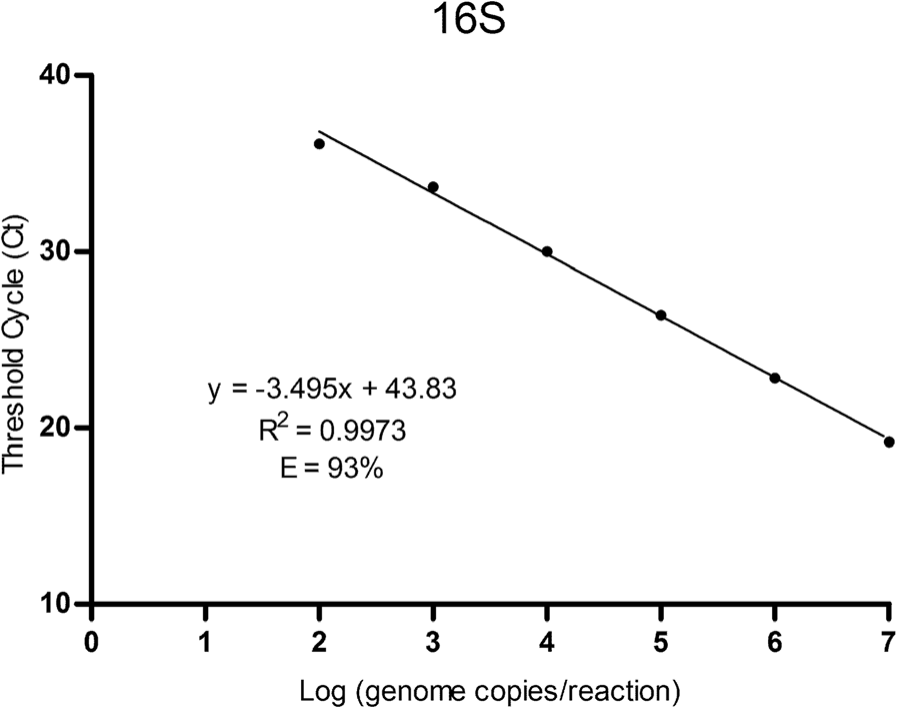
Standard curve of developed TaqMan-MGB real-time PCR for *C. fetus* detection. Each point represents the mean Ct of nine different measures (three independent reactions, three replicates each). The curve equation (y), coefficient of determination (R^2^) and amplification efficiency (E) are indicated

The analytical performance of the assay was determined using a standard curve (Fig. 3). The linear dynamic range of the assay was established between 10^2^ and 10^7^ genome copies per reaction. The amplification efficiency and the coefficient of determination (R^2^) were 93% and 0.9973, respectively. Intra- and inter-assay reproducibility was calculated using the coefficient of variation (CV), which showed considerable low values, being the highest 2.19% (Table 2).

**Table 2:** Intra- and inter-assay reproducibility for the detection of *C. fetus*

Genome copies/reaction	Intra-assay variations		Inter-assay variations	
	Mean Ct (from – to)	CV (from – to)	Mean Ct	CV
1 × 10^1^	-^a^	-	-	-
1 × 10^2^	36.57–37.69	0.97–2.1	37.13	2.19
1 × 10^3^	33.68–34.11	0.48–1.15	33.89	1.05
1 × 10^4^	30–30.07	0.25–0.16	30.03	0.23
1 × 10^5^	26.37–26.46	0.14–0.27	26.41	0.26
1 × 10^6^	22.62–22.86	0.18–0.7	22.74	0.73
1 × 10^7^	19.02–19.23	0.5–0.83	19.12	0.86

*CV* coefficient of variation of Ct values [%]

^a^Ct value out of dynamic range

No fluorescent signal was observed using template DNA from non-*C. fetus* bacterial species used as negative controls (i.e. *C. geochelonis*, *C. hyointestinalis*, *C. jejuni*, *C. coli* and *C. sputorum*). This result corresponds to a clinical specificity of 100 and a 95% confidence interval of 59–100% (Clopper-Pearson interval).

## Discussion

*Campylobacter fetus* is a pathogen of great relevance for the cattle industry and public health. It is mandatory to report the presence *C. fetus venerealis* to the World Organization for Animal Health (OIE). In humans it is necessary to detect this opportunistic pathogen to achieve a better treatment and for epidemiological surveys. Detection of *C. fetus* in humans is difficult because both *C. fetus fetus* and *C. fetus testudinum* are potential pathogens and well-established methods would fail to detect strains of reptilian origin [10]. Therefore, cost-effective, automated and straightforward tools for the unambiguous identification of *C. fetus* are of paramount importance.

Bacteriological analysis, like culture isolation and biochemical tests, are well standardized and extensively used but challenging by the slow growing and few differential phenotypic properties of *C. fetus* [26]. These methods are also laborious and time-consuming, a disadvantage when processing samples at large-scale or delivering a fast diagnosis. To improve the quality and complement the gold-standard bacteriological methods for *C. fetu*s detection, some end-point PCR methods have been designed based on the presence of species-specific amplicons [12, 27–29]; these assays fulfill various criteria such as accuracy, high detection probability and well-standardized protocols for its application and interpretation. Real-time PCR methods have been also designed with the same purpose [14–17] and have provided additional technical improvements to *C. fetus* detection protocols, like the prevention of cross contamination and the minimization of manipulation and running times. However, both end-point and real-time PCR methods described to date are designed to identify *C. fetus* in bovine samples and do not deal with the intra-specific genetic variability of the bacteria that is found in diverse hosts. In comparison to conventional PCR methods, real-time PCR assays provide increased sensitivity and an accurate quantification of target DNA to study the dynamics of the bacteria in different hosts and tissues. To the best of our knowledge, there is not a real-time PCR method that uses ribosomal sequences for the identification and quantification of *C. fetus*. Here, we have improved the current molecular methods for *C. fetus* detection by designing a new real-time PCR assay that targets the multi-copy *16S* rRNA gene. The variability of these sequences within *Campylobacter* species supports its suitability as a target for amplification-based methods using fluorescent probes. The inclusion in the assay of a TaqMan-MGB probe provides higher specificity, sensitivity and accuracy than traditional TaqMan probes and discriminates between sequences that differ in just one nucleotide [30–32].

Our assay was compared to the *cstA* gene end-point PCR proposed by Hum et al. [12] and currently used as standard for molecular diagnosis of *C. fetus*. The bovine sample INTA 89/222 and the reptilian RA8/Italy/2011 could not be detected by Hum’s PCR (Table 1), revealing that the sensitivity of this method for bovine isolates is not complete as previously reported [12, 17, 33–36]. These isolates were confirmed as belonging to *C. fetus* by sequencing a fragment of the *16S*rRNA gene; therefore the lack of amplification of the *cstA* gene could be due to the absence of the target *cstA* gene in these strains, or the presence of sequence variations that prevent the correct annealing of primers. Our attempt to amplify a larger region including Hum’s PCR target region also failed, indicating the absence of this gene in these strains or an even greater sequence divergence within the *cstA* gene (data not shown). To test this hypothesis, it would be necessary to conduct the whole genome analysis of these strains. This notion is supported by the presence of several differences in Hum’s primers binding sites in the complete genome of the reptilian strain *C. fetus* subsp. *testudinum* 03-427 (GenBank Acc. number NC_022759). This explains why the 13 isolates used for the description of this subspecies, and the RA8/Italy/2011 strain analyzed here, were negative for Hum’s method based on the *cstA* gene [10]. Given the importance of this gene in the metabolism of nitrogen, and in the interaction with the host in *C. jejuni* [37], it is necessary to continue investigating its variations and possible roles in *C. fetus*.

Our novel real-time PCR assay detected all *C. fetus* tested in this study, but was negative for other *Campylobacter* species. The complete identity of primer and probe targets in all *C. fetus* strains deposited in the GenBank database (including reptilian isolates) supports that our assay is expected to detect the currently described subspecies from diverse hosts (Fig. 1). These results indicate the excellent sensitivity and specificity of the assay. In addition, the primers and probe sequences are conserved in the *16S*rRNA gene of the three subspecies (Fig. 1), in contrast with what happens with primers that amplify the *cstA* gene.

The assay here described has some advantages over other real-time PCR methods described in the literature. The *nahE* assay reported by Van der Graaf-van Bloois et al. [17] uses a TaqMan probe that provides high sensitivity and detection capability, but its quantification capability has not been ascertained using a standard curve. It is also uncertain whether this assay would detect reptilian *C. fetus testudinum* isolates, for which it was not designed, as the probe and the forward PCR primers have two mismatches each with respect to the *C. fetus testudinum* reference strain 03-427. The hybridization of primers and probes to the *nahE* gene could be also affected because it is embedded in a region that shows genomic rearrangements in most of the complete genome sequences available in the databases (not shown). The methodology to detect the *cpn60* gene described by Chaban et al. [14] uses specific primers and SYBR green chemistry to identify *C. fetus* species, but its performance is sub-optimal in samples with low bacterial concentrations [15], such as the uncultured samples that were successfully tested in the present assay (Table 1).

## Conclusions

The *16S* rRNA gene-targeted assay here developed is excellent for the accurate detection and quantification of *C. fetus* in clinical samples and pure cultures. The possibility to characterize a large number of samples in a rapid, sensitive and reproducible way makes this assays a suitable tool for routine testing and research. For these reasons, this method has the potential to become a new standard in molecular identification of *C. fetus* species.

## Methods

### Real-time PCR design

The assay is based on a set of primers that amplifies a 78-bp sequence of the *16S* rRNA gene (16SFw: 5′-GCACCTGTCTCAACTTTC-3′and 16SRv: 5′-CCTTACCTGGGCTTGAT-3′) and a TaqMan-MGB probe (16SPb: 5′-VIC-ATCTCTAAGAGATTAGTTG-MGB/NFQ-3′), which targets a 19-bp polymorphic region that discriminates strains of *C. fetus* from the remaining *Campylobacter* species and other bacteria. This polymorphic region (Fig. 1) was detected by visual inspection of over 3859 partial and complete *16S* rRNA gene sequences aligned with T-Coffee [38]. The constructed alignment comprised sequences from all recognized *Campylobacter* species and from unassigned strains belonging to the genus, which were obtained from the SILVA database [39]. An alignment of 1907 representative sequences (removing identical sequences) is shown in Additional file 1. BLAST algorithm [40] was used to check *in silico* the specificity of primers and probe sequences, and to evaluate the occurrence of non-specific matches within the genomes of *C. fetus* and other bacterial species.

### Bacterial strains: species and subspecies identification

The real-time PCR assay was tested with a collection of *C. fetus* strains isolated from cattle, humans and reptiles. Two of the strains (INTA 97/C1N3 and INTA 97/608) were assayed also directly from bovine samples of placenta or vaginal mucus, without a previous isolation step. Ten additional strains from four non-*fetus Campylobacter* species that occasionally occur in bovine samples were used to verify the specificity of the assays (Table 1).

Strains were previously typed using bacteriological methods to test the assay specificity. Samples were grown in Brucella semi-solid Broth and *Campylobacter* selective medium under microaerophillic conditions (85% H_2_, 5% O_2_, 10% CO_2_) for 48 h at 37 °C. The presumptive *Campylobacter* colonies were tested by catalase and oxidase tests, and grown in Brucella broth (Sigma-Aldrich, St. Louis, USA) with 1, 1.3, 1.5 and 1.9% glycine (Sigma-Aldrich), without glycine and in Brucella broth with NaCl and cysteine (Sigma-Aldrich) to detect H_2_S production with a lead acetate paper (Sigma-Aldrich). Sodium selenite reduction test was also performed. Colonies that grew in 1% glycine were classified as *C. fetus fetus* or *C. fetus testudinum* by their positive or negative H_2_S production, respectively. Glycine-sensitive colonies were assigned to the subspecies *C. fetus venerealis* (H_2_S negative) or *C. fetus venerealis* bv intermedius (H_2_S positive) (Table 1). Out of a total of 60 strains, 25 were *C. fetus fetus*, 20 *C. fetus venerealis*, 10 *C. fetus venerealis* bv intermedius, one was *C. fetus testudinum*, and four were not analyzed.

Strains were further characterized using the multiplex-PCR assays designed by Hum et al. [12] and Iraola et al. [41]. Both assays use the same species-specific primers to detect the *cstA* gene and different genes to identify the subspecies. The first method includes a fragment of the *parA* gene as a *C. fetus venerealis* marker, and the second uses a fragment of the *virB11* gene (Table 1) [42].

In cases where multiplex-PCR based methods failed to identify the isolates, molecular identification of species was confirmed by sequencing a fragment of the *16S* rRNA gene, which was amplified using the C412F and C1288R primers described by Linton et al [21].

### Real-time PCR assays

DNA was extracted from 500 μL of a suspension of live bacteria in a phosphate-buffered saline pH 7.4 solution (1 × 10^8^ CFU/mL), or from 1 mL of preputial washing or vaginal mucus. The QIAamp DNA Mini Kit (Qiagen, Hilden, Germany) was used for all DNA extractions and the DNA purity was measured as the ratio of absorbance at 260 and 280 nm (A260/280) using a Nanodrop 2000 (Thermo Scientific, Waltham, USA).

Real-time PCR was carried out in a 25-μL reaction containing 1 × TaqMan Genotyping Master Mix (Applied Biosystems, Foster City, USA), 1 × Custom TaqMan SNP Genotyping Assay (0.9 μM each primer and 0.2 μM probe), and 1 μL genomic DNA. Thermocycling was performed on an ABIPrism 7500 (Applied Biosystems) and consisted of a 5 min incubation step at 50 °C, denaturation for 10 min at 95 °C, followed by 40 cycles of 15 s at 95 °C and 1 min at 60 °C, and a final step of 5 min at 70 °C. Fluorescence measurements from VIC fluorophore was collected at the 5 min initial incubation stage, at the 60 °C step of each cycle, and at the end of the run.

### Standard curve generation for analytical testing

To construct the standard curve for the ribosomal probe we generated 10-fold serial dilutions containing 10^0^–10^7^ genome copies/μL. Number of genome copies was determined by the following formula: Y (genome copies/μL) = [*X* (g/μL) DNA/ (nt genome length × 660)] × (6.022 × 10^23^) using the DNA concentration of the dilution (X) and the genome size of the strain Cff 82-40 (1.77 Mb; GenBank accession number NC008599). The log dilution series of *C. fetus* genomes and negative controls containing nuclease-free water were tested with real-time PCR in triplicate and in three independent runs.

Standard curve was generated by plotting threshold cycle (Ct) values per three replicates per standard dilution versus the logarithm of the bacterial genome copies to determine analytical sensitivity and efficiency of the assay. The amplification efficiency was calculated with the equation E = (10^(−1/k)^) − 1, where (k) is the slope of the linear regression line [43, 44]. A value of 1 corresponds to 100% amplification efficiency. The coefficient of determination (R^2^) was also assessed and was considered to be suitable when it was higher than 0.980 in a single run [45, 46]. The coefficients of variation (CVs) of Ct values were assessed separately for each standard bacterial dilution by analyzing the replicates of the same analytical run (intra-assay) and the repeated analyses from different analytical runs (inter-assay).

## Additional files

Additional file 1: Alignment of *16S* rRNA gene sequences from *Campylobacter* species obtained from the SILVA database, in FASTA format. (TXT 4882 kb)
